# Notes and News

**Published:** 1886-10-16

**Authors:** 


### Notes and News.

Collected and Communicated.

H.R.H. the Prince of Wales has given instructions for a further sum of £200, being the amount deposited in Dr. James Aveling's begging-boxes at the Exhibition from July 10th to Oct. 8th, to be forwarded to the Lord Mayor.

Dr. Aveling's begging-boxes have proved a great success, and the hospitals owe him grateful acknowledgments for his happy thought and its skilful application on their behalf.

Prince Albert Victor arrived in Burnley on Tuesday evening, and was the guest of Colonel John Thursby, of Ormerod, who has not only contributed very largely to the Burnley Hospital, but has given the site on which the new buildings have been erected.

On Wednesday morning Prince Albert Victor, after receiving addresses from the Mayor and Corporation, and from the Freemasons of the district, drove through the town, and stayed to luncheon at Bank Hall with Mr. John Thursby, the son of his host. The streets were crowded with people, who warmly cheered the Prince wherever he appeared.

After luncheon he proceeded to the hospital, where a grand reception awaited the Prince, who was evidently gratified and pleased. After the usual formal proceedings and a short speech from Prince Victor, the hospital was duly declared open, and Professor John Marshall, F.R.S., delivered a brief address on the advantages of circular wards in general and the achievements of the good people of Burnley in particular. The whole proceedings passed off without a hitch of any kind, and the greatest credit is due to Colonel John Thursby and to Mr. John Butterworth for their princely liberality, as well as to Mr. Thomas Brown, F.R.C.S., the Committee, and all concerned for the complete success which has crowned their efforts to win for Burnley the legitimate triumph they undoubtedly achieved on Wednesday last.

We must not omit to mention the architects, Messrs. Waddington & Sons, of Burnley, who have shown in this and other recent competitions an exceptional knowledge of hospital requirements and construction. The plans they prepare, so far as we have seen them, invariably exhibit originality of design, skilful arrangement, and a thorough mastery of the requirements of the particular institution for which they are prepared. It would be interesting to know if the original estimates for the Burnley Hospital have been exceeded, and if so, to what extent.

Dr. H. L. Hazlerigg has been appointed one of the honorary medical officers to the Devonshire Hospital, Buxton, *vice* Dr. Flint, resigned.

Miss Rawson, of Nydd Hall, has subscribed the munificent sum of £3,500 towards the cost of the new wing of the Convalescent Hospital at Harrogate.

The Earl of Harewood recently laid the foundation-stone of the new Bath Hospital and Convalescent Home at Harrogate. Lord Harewood and his son, Viscount Lascelles, have given land to extend the site to an area of four acres, and the new buildings, which will be erected at a cost of £15,000, are designed to accommodate 150 patients.

The Harewood family have been patrons of the Bath Hospital ever since its establishment in 1823. It was first opened in 1826. The old buildings are now in process of demolition, and until the new hospital is completed the patients will be accommodated at Cornwall House, which the Committee have leased for two years.

Miss Jeannette Stevens, staff-nurse at St. Bartholomew's Hospital, has been appointed to the position of "Sister" at the Children's Hospital, Pendlebury, near Manchester. Mrs. O'Kell, another staff nurse at the same institution, has been elected Assistant Matron to the St. Helena Home Hospital, St. John's Wood Road.

On the 8th inst. the Earl of Derby visited Manchester to open the new out-patients' department of the Hospital for Consumption and Diseases of the Throat, Hardman Street, with accommodation for 100 patients. The site has cost £3,023, and £1,909 has been expended on the building and furniture. An extension of the hospital is at the same time being carried out at Bowdon, by which the accommodation for patients will be largely increased.

Mr. H. B. Walker has been appointed surgeon to the Lowestoft Hospital, in place of Mr. F. S. Worthington, who has been obliged to resign. The name of Worthington has been associated with this institution since its foundation. The enthusiastic work of the elder Worthington on its behalf has become quite a tradition of the place. It was not, therefore, without a feeling of deep regret that the Governors at their annual meeting last Friday received the resignation of an honoured son of an equally honoured father.

The half-yearly meeting of the Governors of the Devonshire Hospital at Buxton was held on Monday, Dr. Robertson, J.P., presiding. This hospital is suffering from the prevailing depression, and the receipts for the quarter, as compared with the corresponding period of the preceding year, showed a falling-off of £290.

How often do we see the trite adage that truth is stranger than fiction exemplified in every-day life! Some little time since "an accident" was brought into the London Hospital. The unfortunate gentleman, who had been run over by a cab, was carefully carried to the Gloucester Ward, and put to bed. One of the house physicians went to attend the case, when, strange to say, he beheld his own brother!

Two excellent Sunderland institutions, the Children's Hospital and the Infirmary, are to undergo a process of amalgamation. The new building, to be called the "Hartley Memorial Wing," which is to be added to the Infirmary, for the reception of the children, will, it is estimated, cost about £10,000. This is a somewhat formidable sum to raise, but doubtless the men of Sunderland will not be found wanting in so good a work.

The out-patients' department at Westminster Hospital has been closed during the past six months, in order to allow of structural alterations and extensions. The new building for the out-patients, which has just been opened, will prove a vast improvement on the old accommodation. Several other improvements have been carried out at the hospital which will add materially to the comfort of the patients.

The employés of the General Post Office have come forward liberally to the assistance of the Hospital Saturday Fund, the total amount already received from the Post Office exceeding £270. It is expected that the total Post Office collection will considerably exceed last year's amount.

The borough of Derby will soon have a new lunatic asylum capable of accommodating 300 patients. The designs have been prepared by Mr. Jacobs, architect, Hull, and Dr. R. Greene, Medical Superintendent Berry Wood Asylum, Northampton. The amount of the contract is £31,100, and the buildings are now in course of erection.

We are anxious to make The Hospital helpful to convalescent patients and to the philanthropists and officials who take an interest in their welfare. With this object a free employment column has been started, and we shall be happy to receive suggestions, and to publish particulars of individual cases and qualifications, if those interested will communicate with the Editor.

A novel use has been found for postcards in connection with the hospitals. Some of the special hospitals use them with the object of ascertaining how the patients progress after leaving their institution. At one hospital eight hundred and forty-two postcards were received, during the quarter just ended, from patients who had left the hospital for six weeks and upwards. Five hundred and seventy-eight patients represented themselves as improved, two hundred and sixty were no better, and four had died. Of the whole number of patients thus self-reported two-thirds declared themselves to have received permanent benefit.

We are indebted to some unknown friend for a copy of the 1886 report of the Congleton Cottage Hospital. It presents several noteworthy features. The average cost per patient has fallen from £5 3*s.* 11*d.* in 1883, to £4 6*s.* 8*d.* in 1886; the number of patients has, however, been nearly identical in each of these years, and the average number of days in hospital in 1883 was 37½, against 26½ in 1886. We observe that the names of the medical officers are not given in the report, as they certainly ought to be, but the Honorary Secretary is Mr. J. H. Kennerley, and the Matron, Miss Wagnell, "whose skill and attention give the Committee every satisfaction." One lady, recently dead, Mrs. Farrington, had collected some £25 per annum, chiefly in half-crowns, showing what one good woman may accomplish even in a small community. The statement of accounts, the table of cases treated, the number of patients received from each township and village, all prove that there is thought, care, and skill in the management of the Congleton Cottage Hospital.

Mr. Richard Chamberlain, M.P., Mr. G. T. Bartley, M.P., and Mr. Watson Surr, have been appointed to fill vacancies occurring in the Committee of the Great Northern Central Hospital. It is satisfactory to note that this hospital is strengthening its hands by the acquisition of men of influence in the northern district. Too much praise cannot be given to the managers of the institution for their steady and persevering efforts, in the face of untold difficulties, to raise funds for the new building. The present hospital contains about thirty beds, while the new one will contain a hundred and fifty. The district is practically destitute of hospital accommodation. At least fifty thousand pounds will be required, and no more needy or deserving institution can be found in London.

---

Some people seem to possess little feeling, and to be devoid of discretion. A wee mite, Lillian Thompson, *aged three years*, being sent to school, for some alleged offence was put to stand upon a form. What could be expected but that such a baby should get giddy and frightened? She fell off, and, alighting on her head, sustained injuries causing concussion of the brain and death. The coroner justly commented upon the barbarous conduct of the teacher, but the jury returned a verdict that the child's death resulted from a fall. Could anything be sadder or more unsatisfactory?

---

In reference to a note on nurses' certificates from a recognised nursing school, a cottage hospital matron writes: "What constitutes a claim to be a nursing school? It would be unjust to many excellent nurses to narrow this claim to metropolitan, or even to large provincial hospitals. Nurses have excellent opportunities of thorough training in cottage or special hospitals, where of necessity they see and nurse every case, and do much which is elsewhere done by medical students. Could there not be tests of efficiency before granting certificates, by a general examining body, conducted on the lines of the ambulance examinations? That 'certificates' are now, to a great extent, as valueless as private testimonials for proving a standard of efficiency, all must know, who have had experience in the employment of trained certificated nurses, with their varied qualifications. Such a system need in no way alarm those of the medical brotherhood who fear lest nurses, by becoming better educated, should aim at usurping the doctor's functions and powers. Such presumption is the last thing that a sensible, educated woman would desire to show, and this is one of those fears which has proved without foundation, save in a very few isolated cases." What have the nursing schools to say to this suggestion?

---

Miss Kelman, in a recent lecture on nursing, illustrated the need of having the walls of hospitals of a quiet and unobtrusive colour, by telling an anecdote of a patient—an old gentleman—who was heard continually muttering, "Fourteen up, thirty-five across." This fact arrested the attention of the doctor and nurses, who examined the room, and ultimately found that the pattern of the paper was divided into squares of fourteen up and thirty-five down. When the old gentleman fell asleep, he was quietly removed to another room, where, the wall-paper being of a neutral tint, he quickly recovered.

---

It may be asked, with reference to the question of alcohol in hospitals, is it just to give money to certain officials in lieu of beer? This question has been very warmly contested of late at more than one public institution, and the opponents of the system urge that, if such money is to be given at all, then it will not be possible to draw the line at beer, but that a servant may fairly ask for a money advance for each article of diet he or she refrains from consuming. A vegetarian, for example, might with equal justice claim a grant in lieu of meat, and the beer-drinker might ask an equivalent in money value for the milk which the abstainer consumes in large quantities, but of which he partakes but sparsely, if at all. The fact is, a hospital ought to be managed upon business principles; and it is very doubtful if, in the face of the great increase in the consumption of milk, for instance, any hospital committee is justified in making these money allowances at all. Every member of the resident staff is entitled to full board at the hospital cost, and the idiosyncrasies of the individual members are no more to be made the basis of a claim for special money payments, than it would be possible to permit the diverse tastes of the female officials to find expression in the garments they wear whilst on duty. At least, that appears to us to be the business view to take of the matter.

---

West Ham, which is one of the most densely populated portions of the East-end, has proved once more the power of the pence. In the five days ending Saturday, the 9th inst., no less than £903 has been raised at a bazaar, pigeon show, and exhibition, under the chairmanship of Mr. George Hay. During the week more than ten thousand visitors were admitted. Such a result, remembering the circumstances of the district, far exceeds the success attained by any similar entertainment held at the West-end on behalf of a hospital during the current year. There is an enthusiasm about the working classes when they really mean to accomplish a piece of work which is refreshing in its immensity.

